# The applications and mechanisms of *Rosmarinus officinalis* L. in the management of different wounds and UV-irradiated skin

**DOI:** 10.3389/fphar.2024.1461790

**Published:** 2025-01-07

**Authors:** Jianwen Xu, Ting Li, Fei Li, Hong Qiang, Xiaoxiao Wei, Ruiwen Zhan, Yun Chen

**Affiliations:** ^1^ Department of Nursing, Shidong Hospital Affiliated to University of Shanghai for Science and Technology, Shanghai, China; ^2^ Department of Neurology, Shidong Hospital Affiliated to University of Shanghai for Science and Technology, Shanghai, China; ^3^ Special Committee of Scientific Research, Shidong Hospital Affiliated to University of Shanghai for Science and Technology, Shanghai, China; ^4^ Wound Clinic, Department of General Surgery, Shidong Hospital Affiliated to University of Shanghai for Science and Technology, Shanghai, China

**Keywords:** wound healing, UV irradiation, complementary therapy, *Rosmarinus officinalis* L., rosemary

## Abstract

Chronic wounds, especially non-healing wounds, significantly affect patients’ quality of life and raise the costs of therapy. Wound healing is a complicated process involving interdependent stages, which may be impaired and delayed by infections with multi-drug resistant pathogens. Current medical strategies for wound healing, especially the treatment of non-healing wounds, exert limited therapeutic effects, thus become a dramatic challenge for modern medicine. There has been growing interest in exploring complementary approaches to enhance the wound healing process, and complementary therapy using herbs and their related products has gained increasing attention. Apart from skin wounds, dermal pathological changes caused by UV irradiation, may also benefit from such complementary therapy. The antimicrobial, anti-inflammatory, antioxidant, analgesic and collagen-promoting properties of extract from *Rosmarinus officinalis* L. (rosemary) have all been considered to contribute to the beneficial effects on different stages and multiple aspects of skin recovery after various wounds or UV irradiation. This review aims to summarize the applications and their underlying mechanisms of rosemary as part of the complementary therapy for injured and UV-irradiated skin based on the currently available evidence. The medicinal properties of rosemary and its application in wound dressing are first discussed, followed by summarization of its application in different types of wounds. A conclusion is reached and future directions are discussed. As research in this area continue to evolve, rosemary-derived products may become an integral part of holistic wound care strategies, offering a complementary approach to conventional treatments.

## Graphical Abstract

**Figure:**
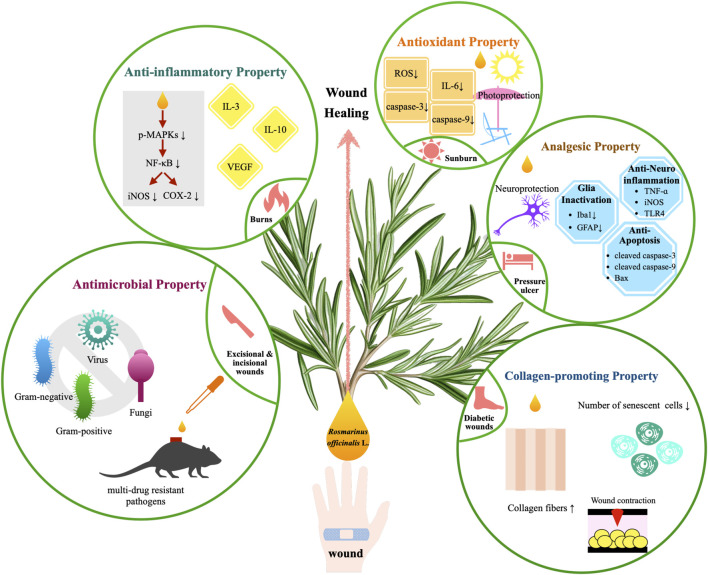
The properties of REO for the enhancement of skin healing process. For the antimicrobial property, REO exhibited activity against Gram-positive, Gram-negative bacteria, virus and fungi, particularly having a high antibacterial potency against MDR pathogens. For the anti-inflammatory property, REO prevented phosphorylation of MAPKs, suppressed the activation of NF-κB and decreased expressions of iNOS and COX-2. The antioxidant activity is associated with direct elimination of ROS and a significant reduction in DNA lesions, caspase-3 and -9 activity and IL-6 secretion. The analgesic property was attributed to its anti-neuroinflammation and neuroprotection, as indicated by the attenuation of markers of glia activation (Iba1, GFAP), inflammatory factors (TNF-ɑ, iNOS, TLR4) and apoptotic mediators (Bax, cleaved caspase-3 and caspase-9) in spinal cords. The collagen-promoting property refers to the enhanced collagen fibers and reduced number of senescent cells, leading to an accelerated wound healing. So far, the application of REO has been tested in a wide range of wounds and UV-irradiated skin, including excisional and incisional wounds, burns, diabetic wounds, pressure ulcer and sunburn.

## 1 Introduction

Wound refers to interruption of the integrity of biological tissues such as the skin and mucous membranes (Kujath and Michelsen, 2008). Various types of wounds are present, and some of the examples include acute wounds such as incisional wounds and skin flaps, chronic wounds such as diabetic wound, pressure ulcer and burns. These wounds are frequently encountered in medical procedures or in daily life. Surgical site infections, which represents infections at or around the incisional site, occurs to around 0.5%–3% of patients undergoing surgeries (Seidelman et al., 2023), and is considered as a major contributor to postoperative morbidity and mortality (Sekhar et al., 2023). Burn injuries are a common type of wounds that occur to around 11 million people worldwide, with 180,000 of which being fatal (WHO, 2018). These injuries may lead to pain, scarring, and disruptions in mental health and quality of life (QoL) (Logsetty et al., 2016; Stone et al., 2016; WHO, 2018; Mason et al., 2019). As a common procedure in medical interventions, the increase of skin flap survival is always a target of interest. Necrosis constitutes around 10%–15% of skin flap cases, which requires additional surgeries and secondary treatments for infections and other associated conditions (Berry et al., 2024). Inadequate perfusion, ischemia/reperfusion injury, excessive cell death and inflammation are major contributors to skin flap necrosis (Fan et al., 2021). Ultraviolet A (UVA) (320–400 nm) is particularly associated with oxidative processes in photoaging, whereas ultraviolet B (UVB) (280–320 nm) is the main cause of DNA damage in UV irradiation. Although UVA accounts for more than 90% of the total UV irradiation to us, UVB is considered as one of the leading environmental risk factors for several dermal pathological changes such as sunburn, erythema, edema and skin cancer (Svobodova et al., 2003; Baek et al., 2017). Chronic wounds, especially non-healing wounds, are reported to dramatically affect quality of life to both patients and their caregivers, and significantly raise the costs of therapy (Stejskalova and Almquist, 2017). Non-healing wounds, which affect 20 million patients annually and require over 31 billion USD each year for treatment (Leaper et al., 2015), are particularly prone to infections caused by antibiotic-resistant microorganisms embedded within an extracellular matrix (ECM) biofilm communities, resulting in chronic inflammation that delays wound healing (Lipsky and Hoey, 2009; Percival et al., 2012; Bowler, 2018). The 5-year mortality rate for individuals with diabetic non-healing wounds (30.5%) is comparable to that for patients with cancer (31%) (Armstrong et al., 2020). Together, these wounds may affect the effect of medical treatments, as well as interfering individuals’ health outcome and daily life, highlighting the necessity to optimize wound healing.

Wound healing is a complicated natural regeneration process after injury, aiming at minimizing or eliminating scarring as well as promoting damage repair. Different interdependent stages, including coagulation, inflammation, proliferation and remodeling, are involved in the healing process (Eming et al., 2007). The wound care strategies, especially the treatment of non-healing wound is a dramatic challenge for modern medicine. Current strategies for eradicating wound biofilms, which involve removal of infected tissues and topical application of wound dressing with antiseptic agents, exert limited therapeutic effects (Kaiser et al., 2021). Hence, there has been a growing research interest in finding complementary therapies to enhance the healing process. Complementary therapy, which is defined as the use of natural remedies in addition to mainstream medicine, have been increasingly tested in different medical applications and shown to improve patients’ quality of life and human’s wellbeing (Buckle et al., 2014). Traditional remedies, especially plant-based formulations, have been demonstrated to promote wound healing in an impressive way by participating in single or multiple stages during the healing cascade and exhibiting a broad spectrum of antimicrobial activity against Gram-positive, Gram-negative bacteria and fungi (Agreles et al., 2021).

Essential oils are known as unstable volatile and lipophilic liquid or semiliquid products extracted from nonwoody parts of the plants (flowers, seeds, leaves, fruits and roots) as their secondary metabolites, and are mainly composed of low-molecular-weight organic compounds such as terpenes, terpenoids, phenylpropanoids, aromatics and short-chain aliphatics (Ait-Ouazzou et al., 2011). Due to the chemical properties of their constituents and sequential chemical and enzymatic reactions during the handling processes, essential oils can be easily degraded when exposing to heat, humidity, oxygen and light, which may impair their biological activities. For instance, topical application of essential oils containing oxidized terpenoids may have skin-sensitizing effects to induce allergic contact dermatitis (Brared Christensson et al., 2009). In recent years, more and more work has been reported to develop nanoencapsulation of the active compounds of essential oils, which may provide an efficient strategy for preventing their degradation, improving skin contact and permeation, thus protecting bioavailability and beneficial effects (Montenegro et al., 2017; Mofazzal Jahromi et al., 2018; Khezri et al., 2019; Ibrahim et al., 2022).

Notably, promising benefits in the healing process of different wounds and UV-irradiated skin have been shown from one of the commonly used essential oils, which is derived from the aromatic herb *Rosmarinus officinalis* L., a woody perennial herb native to the Mediterranean region, also known as rosemary (Raskovic et al., 2014). Phytochemical analysis revealed that the most abundant bioactive molecules in rosemary included monoterpenes such as 1,8-Cineole, ɑ-pinene and β-pinene, diterpenes such as carnosic acid and carnosol, triterpenes such as oleanolic acid and ursolic acid, flavonoids such as luteolin and genkwanin, and phenolic acids such as rosmarinic acid and caffeic acid (Francisco et al., 2020), many of which have been revealed to exhibit beneficial effects such as antimicrobial and anti-inflammatory effects. To date, ongoing studies have been carried out exploring the application of *R. officinalis* L. in the healing process of acute and chronic wounds and UV-irradiated skin due to its antimicrobial, anti-inflammatory, antioxidant, analgesic and collagen-promoting properties. This narrative review aims to summarize the research updates of rosemary’s application in enhancing the healing process of different wounds and UV-irradiated skin, including the results from clinical trials and laboratory studies, and propose a future research direction in this area. We first introduce rosemary essential oil (REO), including its antimicrobial, anti-inflammatory, antioxidant, analgesic and collagen-promoting properties, as well as its utilization in wound dressing. Then we move on to discuss its application to acute wounds including excisional and incisional wounds as well as skin flap survival, chronic wounds including diabetic wounds, pressure ulcer and burns, and UV-irradiated skin. Lastly, a conclusion is made, and future directions are discussed.

## 2 Methods

A literature search was performed on the global databases (PubMed, Google Scholar, Web of Science), using the keywords “rosemary” and “wound” or “skin”, published in English. For PubMed specifically, the search strategy was [rosemary (Title/Abstract)] AND {[wound (Title/Abstract)] OR [skin (Title/Abstract)]}. A total of 106 English articles were further assessed by independent authors and selected in the order of title, abstract and content of the articles. This assessment also excluded reviews, systematic reviews, meta-analyses, commentary and prospectives. Additional articles from the selected ones were also included in the final manuscript based on their relevance to the topic. Literatures were reviewed independently by two authors. When discrepancies were present, a discussion was performed, which involved a third author who had sufficient expertise in the relevant fields, and an agreement was reached following the discussion.

## 3 Medicinal properties of rosemary essential oil and its application in wound dressing

**FIGURE 1:**
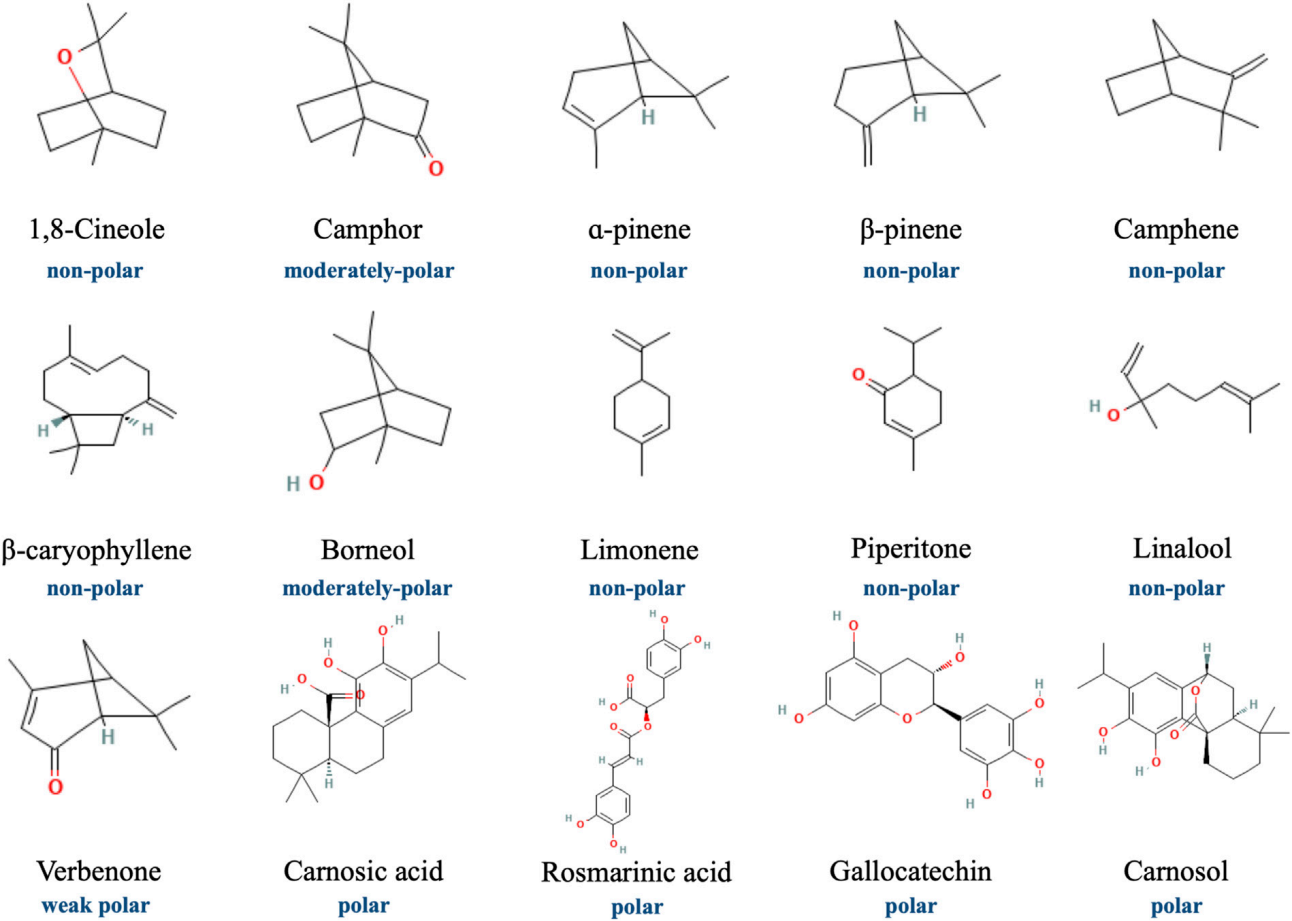
Chemical structure depictions of main compounds in REO (from PubChem).

REO contains a complex mixture of bioactive phytochemicals, which are the sources of its balsamic, camphoraceous and slightly minty odor. Variations in oil composition of rosemary have already been described in different studies (Table 1; Figure 1), which may be affected by a wide range of differences in climate conditions, geographic locations, seasons of harvesting, part of the plant used for extraction, processing of the plant before extraction, extraction techniques, etc. In folk medicine, rosemary has shown its beneficial effects in the treatment of a variety of conditions, including headache, epilepsy, poor circulation, etc., (Yu et al., 2013). For its application in skin health, REO can be used as a massage oil to produce hyperemia and as a component in cosmetic formulations to treat cellulite, alopecia, ultraviolet damage and ageing (Guzman and Lucia, 2021). Its wound healing effects may be enhanced and the skin hydration and elasticity may be improved when using REO loaded by lipid nanoparticles, which were developed on the basis of natural lipids (e.g., cocoa butter as solid lipid, and olive oil or sesame oil as liquid lipids) (Montenegro et al., 2017; Saporito et al., 2018). The antimicrobial, anti-inflammatory, antioxidant, analgesic and collagen-promoting properties of REO have been attributed to a wide range of chemical compounds derived from the plant extract, which have consequently been addressed to enhance the healing process of different wounds and UV-irradiated skin. For instance, 1,8-cineol and ɑ-pinene are key compounds for the antimicrobial activity especially against *Pseudomonas aeruginosa* and *Staphylococcus aureus* (Khezri et al., 2019; Melkina et al., 2021). Additionally, 1,8-cineol, ɑ-pinene and camphor have been reported to exert beneficial effects on different wounds (Khezri et al., 2019) and skin slap survivability (Ince et al., 2018), via their anti-inflammatory properties. Notably, the collagen-promoting property of camphor has been verified in human primary dermal fibroblasts in a dose- and time-dependent manner, as well as in UV-exposed mouse skin (Tran et al., 2015). Meanwhile, the antioxidant and collagen-promoting properties were also found to be attributed to two phenolic diterpene found in rosemary, namely carnosol (Yeo et al., 2019) and carnosic acid (CA) (Park et al., 2013), respectively, while the analgesic property was often achieved by a polyphenol compound rosmarinic acid (RA) (Ghasemzadeh Rahbardar et al., 2017).

**TABLE 1:** Percentages of the main volatile constituents in REO derived from leaves of *Rosmarinus officinalis* L. and their main functions.

Compounds	Geographical origin	EO preparation	Extract preparation/solvent	Analytical Techniques	Percentage in extract (%)	Ref	Main functions
1,8-cineole	Poland	Commercialized source	—	GC-FID-MS	46.4	Sienkiewicz et al. (2013)	As a main antibacterial compound in REO (Khezri et al., 2019) As a main contributor to the skin flap survival (Ince et al., 2018)
	Greece	Steam distillation	Maceration/38% v/v ethanol	GC-MS LC-MS	40.10	Christopoulou SD (2021)	
	China	Steam distillation	—	GC-MS	26.54	Jiang et al. (2011)	
	Iran	Steam distillation	Maceration/water	GC GC-MS	7.43	Gachkar et al. (2007)	
	Italy	Steam distillation	—	GC-MS	7.26	Sacchetti et al. (2005)	
	China	Steam distillation	—	GC-MS	27.23	Wang et al. (2008)	
	Morocco	Steam distillation	—	GC-MS	43.99	Ait-Ouazzou et al. (2011)	
camphor	Poland	Commercialized source	—	GC-FID-MS	11.4	Sienkiewicz et al. (2013)	In human dermal fibroblasts, induce cell proliferation and protect cell senescence (Tran et al., 2015) In UV-exposed mouse skin, maintain collagen level, prevent thickening of epidermis and maintain skin elasticity (Tran et al., 2015)
	Greece	Steam distillation	Maceration/38% v/v ethanol	GC-MS LC-MS	12.40	Christopoulou SD (2021)	
	China	Steam distillation	—	GC-MS	12.88	Jiang et al. (2011)	
	Italy	Steam distillation	—	GC-MS	14.6	Sacchetti et al. (2005)	
	China	Steam distillation	—	GC-MS	14.26	Wang et al. (2008)	
	Morocco	Steam distillation	—	GC-MS	12.41	Ait-Ouazzou et al. (2011)	
ɑ-pinene	Poland	Commercialized source	—	GC-FID-MS	11.0	Sienkiewicz et al. (2013)	As a main antibacterial compound (Khezri et al., 2019) In bacterial cells, to induce the formation of ROS and cause bacterial DNA damage and increase membrane permeability (Melkina et al., 2021) As a main contributor to the skin flap survival (Ince et al., 2018)
	Greece	Steam distillation	Maceration/38% v/v ethanol	GC-MS LC-MS	12.94	Christopoulou SD (2021)	
	China	Steam distillation	—	GC-MS	20.14	Jiang et al. (2011)	
	Iran	Steam distillation	Maceration/water	GC GC-MS	14.9	Gachkar et al. (2007)	
	Italy	Steam distillation	—	GC-MS	6.65	Sacchetti et al. (2005)	
	China	Steam distillation	—	GC-MS	19.43	Wang et al. (2008)	
	Morocco	Steam distillation	—	GC-MS	10.09	Ait-Ouazzou et al. (2011)	
β-pinene	Poland	Commercialized source	—	GC-FID-MS	9.2	Sienkiewicz et al. (2013)	Not mentioned
	Greece	Steam distillation	Maceration/38% v/v ethanol	GC-MS LC-MS	8.94	Christopoulou SD (2021)	
	China	Steam distillation	—	GC-MS	6.95	Jiang et al. (2011)	
	Italy	Steam distillation	—	GC-MS	0.69	Sacchetti et al. (2005)	
	China	Steam distillation	—	GC-MS	6.71	Wang et al. (2008)	
	Morocco	Steam distillation	—	GC-MS	4.84	Ait-Ouazzou et al. (2011)	
camphene	Poland	Commercialized source	—	GC-FID-MS	5.2	Sienkiewicz et al. (2013)	Not mentioned
	Greece	Steam distillation	Maceration/38% v/v ethanol	GC-MS LC-MS	6.38	Christopoulou SD (2021)	
	China	Steam distillation	—	GC-MS	11.38	Jiang et al. (2011)	
	Italy	Steam distillation	—	GC-MS	2.29	Sacchetti et al. (2005)	
	China	Steam distillation	—	GC-MS	11.52	Wang et al. (2008)	
	Morocco	Steam distillation	—	GC-MS	3.82	Ait-Ouazzou et al. (2011)	
β-caryophyllene	Poland	Commercialized source	—	GC-FID-MS	3.5	Sienkiewicz et al. (2013)	Not mentioned
	China	Steam distillation	—	GC-MS	2.37	Jiang et al. (2011)	
	China	Steam distillation	—	GC-MS	2.41	Wang et al. (2008)	
borneol	Poland	Commercialized source	—	GC-FID-MS	3.1	Sienkiewicz et al. (2013)	Not mentioned
	China	Steam distillation	—	GC-MS	3.06	Jiang et al. (2011)	
	Italy	Steam distillation	—	GC-MS	10.4	Sacchetti et al. (2005)	
	China	Steam distillation	—	GC-MS	3.17	Wang et al. (2008)	
limonene	Poland	Commercialized source	—	GC-FID-MS	1.0	Sienkiewicz et al. (2013)	In bacterial cells, to induce the formation of ROS and cause bacterial DNA damage and increase membrane permeability (Melkina et al., 2021)
	China	Steam distillation	—	GC-MS	1.32	Jiang et al. (2011)	
	China	Steam distillation	—	GC-MS	1.95	Wang et al. (2008)	
piperitone	Iran	Steam distillation	Maceration/water	GC GC-MS	23.7	Gachkar et al. (2007)	Not mentioned
linalool	Poland	Commercialized source	—	GC-FID-MS	0.5	Sienkiewicz et al. (2013)	Not mentioned
	Iran	Steam distillation	Maceration/water	GC GC-MS	14.9	Gachkar et al. (2007)	
	Italy	Steam distillation	—	GC-MS	2.18	Sacchetti et al. (2005)	
	China	Steam distillation	—	GC-MS	0.25	Wang et al. (2008)	
verbenone	Italy	Steam distillation	—	GC-MS	21.76	Sacchetti et al. (2005)	Not mentioned
	China	Steam distillation	—	GC-MS	1.48	Wang et al. (2008)	

### 3.1 Properties of REO

#### 3.1.1 Antimicrobial property

The antibacterial property of rosemary extract have been shown against both Gram-positive (*Staphylococcus* and *Bacillus* spp.) and Gram-negative (*Salmonella* and *Campylobacter* spp.) bacterial strains (Santoyo et al., 2005; Bozin et al., 2007; Klancnik et al., 2009; Jiang et al., 2011). Some studies identified the major components of these extracts with varied polarity (Figure 1), namely ɑ-pinene, 1,8-cineole, camphor, borneol, verbenone, limonene, Z-linalool oxide, as potential contributors to the antimicrobial effects (Santoyo et al., 2005; Bozin et al., 2007; Jiang et al., 2011), while others considered CA and RA as the compounds for antimicrobial activity (Klancnik et al., 2009; Zhong et al., 2021). Therefore, rosemary extract that contains gallocatechin, RA and luteolin-3-O-acetyl-O-glucuronide as major components and essential oil that contains 1,8-cineole, camphor, ɑ-pinene, β-pinene and camphene as major components have been suggested to be served as health-promoting ingredients in drinks (Christopoulou SD, 2021). After exposing the *Staphylococcal* biofilms that mimics the condition of chronic wound milieu to REO that presents ɑ-pinene, 1,8-cineole and camphor as major components, a high reduction of 83% of biofilm-forming strains was yielded (Brozyna et al., 2024). Specifically, the polar compounds of rosemary, which includes CA and carnosol, have been identified to have more potent antibacterial activity against *S. aureus* (*S. aureus*) and *E. coli* (*E. coli*), as compared with other compounds (Zhong et al., 2021). The antibacterial activity against *S. aureus* and wound healing benefits of REO has also been demonstrated in Wistar rats with *S. aureus*-infected incisional wounds compared to controls without such treatment (Izadpanah et al., 2017). Apart from *S. aureus* and *E. coli*, rosemary extract, especially its methanol extract (Hofling et al., 2010), seems to have a potent anti-fungi property against *Candida albicans*, via suppressing the fungi’s production of protease to protect against its invasion and adherence to the host tissue and subsequent plaque formation (Smullen et al., 2012). In several *in vivo* studies, topical application of REO on Wistar rats with wound infections accelerated the wound healing rate compared to controls, possibly in a dose-dependent manner, since treatment with cream containing 4% rosemary oil showed a significantly better outcome compared to that using cream containing 2% rosemary oil (Nejati and Nagadehi, 2015; Farahpour MR et al., 2017). Some key ingredients of REO, such as pinene and limonene, were demonstrated to damage bacterial DNA and proteins (e.g., heat shock proteins) and increase membrane permeability following inducing the formation of reactive oxygen species (ROS) in bacterial cells, to contribute to the antibacterial activity (Melkina et al., 2021).

Microbial wound colonization (typically due to *S*. *aureus*, *P. aeruginosa*, *Streptococcus pyogenes* and *Clostridium* strains), as well as bacterial replication in the wound site has been proved responsible for a further delay in wound healing (Bowler et al., 2001). The use of topical antimicrobial agents, e.g., silver sulfadiazine, may reduce the risk of infection, however, microbial resistance to antibiotics causes public health problems. The risk of non-healing wound infected with multi-drug resistant (MDR) pathogens has brought the wound care management into a “chaos” (Khalil et al., 2022). The advantages of essential oils over other antimicrobial agents lies on their broad antibacterial spectrum without introducing the mechanisms of antibacterial resistance. Several phytochemical components from REO have been shown to contribute to its beneficial effects in antimicrobial properties. The acetonic extracts of rosemary, which contains thiocyanic acid, phenylmethyl ester and a wide range of other components, have been demonstrated to have a high antibacterial potency against MDR pathogens in skin burn infections (Khalil et al., 2022). Moreover, REO that contains 1,8-cineole, camphor, ɑ-pinene, β-pinene as well as other major components has also been shown to exert additive or synergistic effects when applying as an add-on therapy for recommended antibiotics, such as cefquinome and sulphquinoxaline, to patients with difficult-to-treat infections (Sienkiewicz et al., 2017). In a wound mouse model infected with *S*. *aureus* and *P. aeruginosa*, topical application of REO promoted healing process by lowering tissue edema, bacterial growth and colonization on the first few days after wound creation, and elevating interleukin (IL)-3, IL-10 and vascular endothelial growth factor (VEGF) levels, neovascularization, collagen deposition, fibroblast infiltration and re-epithelization in the 7 days after the wound creation (Khezri et al., 2019). As reported in previous studies, IL-3 (Yogesha et al., 2009) and IL-10 (King et al., 2014) have anti-inflammatory property and reduce tissue edema in wound tissues, whereas VEGF promotes cell migration, proliferation and ECM protein synthesis to fasten wound closure (Manzuoerh et al., 2019). The potential molecular therapeutic mechanism of REO against *P. aeruginosa* has been associated with its inhibition of elastase, which is produced by this bacteria as well as skin and neutrophils (Sivamani et al., 2012).

In a previous *in vitro* study, Rosemary is the only potent inhibitor of oral *Streptococci* among the twelve tested herbs (Tsai et al., 2008). When investigating forty male adult mixed-breed rabbits inducing a typical oral mucosal wound, topical application of an Orabase paste (comprising olive oil and beeswax) formation containing a mixture of 25 µL/100 g rosemary extract oil, 0.25 mL/100 g hyaluronic acid and 0.04 g/100 g pure metronidazole powder had a better healing response by inhibiting inflammation, achieving faster re-epithelialization and reducing wound contraction, as compared to the topical use of Orabase paste only (Abid and Naser, 2021).

#### 3.1.2 Anti-inflammatory property

The anti-inflammatory effects of REO have been reported in multiple studies (Peng et al., 2007; Takaki et al., 2008). Regarding the mechanisms of how rosemary prevents inflammation, Yu and colleagues found that the methanol extract of rosemary and its hexane fractions prevented the phosphorylation of mitogen-activated protein kinases (MAPKs), thereby suppressed the activation of nuclear factor kappa-light-chain-enhancer of activated B cells (NF-κB), which in turn caused decreased expressions of inducible nitric oxide synthase (iNOS) and cyclooxygenase-2 (COX-2), two important enzymes for mediating inflammatory processes by synthesizing NO and PGE_2_, respectively (Yu et al., 2013). This was consistently observed in another study in which purified extracts from fresh leaves of rosemary and its purified compounds CA and carnosol was applied on mouse skin (Mengoni et al., 2011).

#### 3.1.3 Antioxidant property

Rosemary is a potent source of natural antioxidants, which has been reported in multiple studies (Bozin et al., 2007; Christopoulou SD, 2021). Almost 90% of the antioxidant activity of rosemary leaf extract can be attributed to carnosol and CA (Lo et al., 2002). The antioxidant property of rosemary extract, which is predominantly mediated by oxygenated monoterpenes in REO (Labib et al., 2019), is particularly important to its photoprotective and anti-wrinkle effects, since the antioxidant activity is associated with direct elimination of ROS that prevents oxidative damage of cellular molecules and apoptosis, and is also linked to a significant reduction in DNA lesions, caspase-3 and -9 activity and IL-6 secretion (Vostalova et al., 2010).

#### 3.1.4 Analgesic property

Neuropathic pain is caused by diseases or injuries affecting the somatosensory system, which involves the activation of nociceptive pathways and abnormal responses to noxious (hyperalgesia) or innocuous (allodynia) stimuli (Jensen et al., 2011). Neuropathic pain is probably one of the most common and distressing symptoms in patients with diabetic peripheral neuropathy with a prevalence of 16%–26% (Davies et al., 2006; Abbott et al., 2011). The ethanolic extract of aerial parts of rosemary, as well as rosmarinic acid, have demonstrated as potential remedy in treating neuropathic pain in a rat model through modulating neuroinflammation (Ghasemzadeh Rahbardar et al., 2017), as well as in exerting peripheral analgesic effects *in vivo* (Lucarini et al., 2013). When blending REO with other four essential oils (including geranium, lavender, eucalyptus and chamomile) at a ratio of 1:1:1:1 in a 5% solution and applied to diabetic patients with neuropathy in the aromatherapy massage sessions for 4 weeks, both symptoms of neuropathic pain and patients’ quality of life have been improved compared to controls (Gok Metin et al., 2017), providing further evidence for the analgesic property of REO. A previous study showed that the beneficial effects of rosemary alcoholic extract administered at 400 mg/kg on neuropathic pain in rats was attributed to its anti-inflammatory property, as indicated by the attenuation of markers of glia activation (Iba1, GFAP), inflammatory factors (TNF-ɑ, iNOS, toll-like receptor 4) and apoptotic mediators (Bax, cleaved caspase-3 and caspase-9) in spinal cords (Ghasemzadeh et al., 2016).

#### 3.1.5 Collagen-promoting property

As a key component of ECM, collagen plays a critical role in wound healing by providing structural support to the healing tissue, therefore, organizing and strengthening collagen fibrils could fasten wound contraction and recovery (Tracy et al., 2016). Fibroblasts trigger wound contraction by the synthesis of ECM proteins such as collagen (Shinde et al., 2017). As one of the major constituents of REO, camphor has been demonstrated in human dermal fibroblasts to not only induce cell proliferation via the activation of PI3K/Akt and Erk signaling pathways, followed by the Akt-triggered upregulated expression of collagen type 1 at the protein, mRNA and promoter levels, but also protect cells from senescence, as indicated by a reduced number of senescence-associated β-galactosidase-positive cells (Tran et al., 2015). Additionally, camphor has been shown a significant effect on the skin recovery of mice following a 4-week UV exposure compared to controls, via preventing UV-induced collagen degradation, thickening of the epidermis and subcutaneous fat layer, as well as loss of elastin (Tran et al., 2015). Such UV-protective effects have also been reported when applying CA, a phenolic diterpene of rosemary, on human dermal fibroblasts and keratinocytes, via attenuating Erk/AP-1 pathway and ROS generation (Park et al., 2013).

### 3.2 REO application in wound dressing

Although wound dressing materials (e.g., hydrogels) are commercially available (Peng et al., 2022), plant-based wound dressing is gaining its popularity in wound care management due to inherent medicinal properties and environmental sustainability, reduced toxicity and side effects, as well as increased ease of accessibility for cost-cutting (Ali et al., 2020). For instance, a novel wound dressing with rosemary extract composited with carbon quantum dots (CQDs) and Fe_(3)_O_(4)_ loading onto poly vinyl alcohol (PVA)-cellulose nanofibrils has been tested recently, and yielded a good application prospect due to its appropriate mechanical properties, prolonged release, limited toxicity and improved cell proliferation and migration ability (Rooholghodos et al., 2023). For another instance, when REO composited with adsorbed silver (Ag) nanoparticles was encapsulated in polyurethane (PU)-cellulose acetate electrospun fibers, which were produced from food-grade biopolymer (Hosseini et al., 2021), and the encapsulated REO showed an impressive antibacterial activity against *S. aureus* and *E. coli*, along with an improved hydrophilicity of the fibers for a better cell attachment, making it a desirable wound dressing material for actual clinical application (Rather et al., 2023). Previous *in vitro* and *in vivo* research showed that incorporation of 0.25–0.75 wt% powdered rosemary ethanolic extract into polylactic acid (PLA) improved the biocompatibility of the extracted product and enhanced the antibacterial and antioxidant properties at the same time (Darie-Nita et al., 2018).

## 4 REO application in different wound types

The application of REO in a variety of wound types have been studied in different animal models, however, evidence from clinical trials is still limited, which is summarized in Table 2.

**TABLE 2:** Clinical evidence of the safety and effectiveness of rosemary-derived products on the treatment of wound and UV-irradiated skin.

Wound	Participants/groups	Intervention Description	Evaluations	Conclusion	Ref
Acute Wounds					
Episiotomy wound	Primiparous women 1. Intervention (rosemary cream) (n = 46) 2. Control (placebo) (n = 46)	Prescribed cream was topically applied on the sutured area 1. Dose: 2 cm 2. Frequency: twice daily 3. Duration: 10 consecutive days postpartum	REEDA scale (12h, day 4, day 10)	Safe and effective	Hadizadeh-Talasaz et al. (2022)
Burns	Two children with burn injuries (case study)	A combined essential oil formulation containing rosemary was topically applied	Not applicable	Had a shorter length of PICU and hospital stays, no blood stream infections	Jopke et al. (2017)
Chronic Wounds					
Pressure ulcer	Patients with pressure ulcer (n = 70) 1. Intervention (rosemary ointment) 2. Control (routine care)	1. Dose: not mentioned 2. Frequency: once daily 3. Duration: 7 days	Pressure Ulcer Scale for Healing (before, day 3 and day 7 after)	Rosemary facilitated healing and prevented progression of grade I press ulcers	Khoshoei Parizi and Heidari (2022)
UV-irradiated Skin					
Sunburn	Healthy volunteers (n = 10) only intervention	Oral ingestion of rosemary extracts was provided to all participants 1. Dose: 250 mg of NutroSun^®^ 2. Frequency: once daily 3. Duration: 12 weeks	Minimal erythema dose (MED)	A significant MED was increased after 8 weeks, stronger protection was achieved after 12 weeks	Perez-Sanchez et al. (2014)

### 4.1 Acute wounds

#### 4.1.1 Excisional and incisional wounds

Animal studies revealed that REO may present beneficial effects on healing of excisional and incisional wounds. In an excision wound rat model, the topical application of REO exerted beneficial effects on wound healing including improved wound contraction and re-epithelialization with activated hair follicles, which was further enhanced by using a mixture containing 10% v/v tea tree (*Melaleuca alternifolia*) and rosemary (*R. officinalis* L.) oils compared to untreated controls (Labib et al., 2019). The healing activity of skin lesions have also been observed in a study in which topical application of ointment consisting of 10% rosemary-of-Chapada essential oil has been performed on rats, showing that the ointment accelerates the initial stages of healing, reduces inflammation, increases collagen fibers density, and promotes angiogenesis and wound contraction compared to controls (Bulhoes et al., 2022). Additionally, the effectiveness of topical application of REO in healing skin incisional wounds has also been proved in mouse (de Araujo et al., 2017) and rat (Izadpanah et al., 2017) models.

Few clinical studies have been performed evaluating the beneficial effects of REO on excisional and incisional wound healing. Existing randomized controlled trial applying 3% rosemary cream to primiparous pregnant women who received episiotomy during the late second stage of labor revealed the effectiveness of rosemary extract on episiotomy wound healing, with a higher rate of healing compared to controls (Hadizadeh-Talasaz et al., 2022). Additionally, the safety of rosemary application on pregnant women has also been testified, since no complication (fever, shivering, sensitivity to the cream in general or in the wound area, severe pain, swelling, burning sensation, itching, stiffness, dryness, and purulent discharge in the wound area) was reported by mothers in the intervention group during the whole study (Hadizadeh-Talasaz et al., 2022).

#### 4.1.2 Skin flap survival

Skin flaps are frequently utilized in soft-tissue reconstruction. However, necrosis is often seen as a complication that affects skin flap recovery. To solve this problem, a group in Turkey has performed a series of experiments on rats to evaluate whether rosemary extracts could prevent flap necrosis. In their initial attempts, they confirmed that both local use of REO by topical application and systemic use of REO by oral administration exerted vasodilatory effects, resulting in increased blood flow to the flap and reduced occurrence of dreaded necrotic complications compared to controls (Ince et al., 2015; Ince et al., 2016). This group has also identified two bioactive compounds from REO, ɑ-pinene and cineole, as the main systemic contributors to the improved flap survival (Ince et al., 2018).

### 4.2 Chronic wounds

#### 4.2.1 Diabetic wounds

Diabetic wounds pose a significant challenge in healthcare. Delayed cutaneous wound repair is a chronic complication in diabetic patients, which is resulted from a variety of factors including hyperglycemia, compromised immune function, oxidative stress, vascular insufficiency, infections and deregulated apoptosis (Rai et al., 2005; Hirsch et al., 2008). Several other diabetic complications, such as neuropathy, nephropathy, atherosclerosis and foot deformities may worsen the diabetic wounds and develop to ulceration, necrosis and even amputation (Hirsch et al., 2008). Animal studies showed that bioactive extracts from REO contributed to healing of diabetic wound via different mechanisms. Polyphenols including RA and CA, which are main bioactive ingredients in rosemary, have shown fasting-glucose lowering effects with a prebiotic effect on gut microbiota (increased amount of diabetes-resistant bacteria such as *Actinobacteria*, *Bacteroides*, *Faecalibacterium*, *Lachnospiraceae*, *Prevotella*, and decreased number of diabetes-sensitive bacteria such as *Firmicutes* and *Ruminococcaceae*) and a notable reduction of oxidative stress in streptozotocin (STZ)-induced diabetic rats, after daily intragastrical administration for 8 weeks (Ou et al., 2018). In the same diabetic rat model, rosemary extracts have also been shown to exert antihyperalgesic and neuroprotective effects in contrast to the persistently decreased nociceptive threshold and increased motor deficits observed in non-treated diabetic animals, which may be partially explained by the inhibition of neuronal apoptosis when administered at 200 mg/kg (Rasoulian et al., 2019). Furthermore, the topical application of 10% rosemary aqueous extract and REO administered at 25 µL per excision wound accelerated wound healing in both diabetic rats (Umasankar and Backyavathy, 2012) and mice (Abu-Al-Basal, 2010) compared to antibiotic treated or untreated controls, via reducing the levels of inflammatory cytokines and promoting collagen production for the improvement of structural integrity of the healing tissue. However, clinical evidence for the potential benefits of REO in diabetic wound care is lacking, therefore, future clinical trials should focus on incorporating it into the treatment regime, to establish optimal dosage, duration, form of application, long-term efficacy and safety.

#### 4.2.2 Pressure ulcer

Pressure ulcer, which is frequently seen in bedridden patients and generates a worldwide concern for healthcare system, could be a result of the continuous pressure with or without shear on the skin and underneath soft tissue. The result of a clinical trial in the intensive care unit showed that application of 30 g tube of rosemary ointment that contains 2.4 g rosemary oil led to decreased scores of the Pressure Ulcer Scale for Healing (PUSH), in contrast to unchanged scores in routine care group, facilitated the healing process and prevented the progression of grade I pressure ulcers (Khoshoei Parizi and Heidari, 2022).

#### 4.2.3 Burns

Burns are skin wounds affecting a significant portion of the body, with wound pain, infection, inhalation injury, pneumonia, scarring, debriding and reconstructive procedures, pruritis and mental health instability as common complications resulted from burn injuries (Wolf et al., 2014). As reported, hospital admissions related to burn injuries reach 40,000 per year, and patients with extensive burns (defined as greater than 30% total body surface area) often require longer hospital stay. This raised the risk of hospital acquired conditions, particularly infections with *Pseudomonas*, *Acinetobacter* and *S. aureus*, which can impede the normal wound healing process. Therefore, prevention and treatment of microbial infection is one of the key factors for clinical management of burn injuries (Mofazzal Jahromi et al., 2018). Animal studies suggested a potential role REO plays in healing of burns. Rosemary extract at a concentration of 1.5%–3% or its isolated compounds at 2–128 μg/mL has demonstrated superior antimicrobial effect against *S. aureus* compared to controls or lower concentrations in multiple studies (Izadpanah et al., 2017; Zhong et al., 2021). The 5% w/w encapsulated REO and the mixed herbal ointment containing rosemary extracts have enhanced skin wound healing on rat burn models (Saporito et al., 2018; Farhan et al., 2021). Additionally, using the mixture of *Thymus vulgaris* honey and REO possessed the highest wound healing rate (89.65%) at day 14 in chemical-induced burn model of rabbits, as compared to *T. vulgaris* honey only (67.5%) or combined with essential oils of *Origanum vulgare* (77.36%) or *T. vulgaris* (82.14%) (Mekkaoui et al., 2021).

Limited number of clinical study has been performed assessing the beneficial effects of rosemary in healing of burns. In a case study, the two children who experienced burn injuries had a shorter length of PICU and hospital stays and did not develop any blood stream infections after using a combined essential oil formulation containing rosemary (Jopke et al., 2017), indicating that the therapeutic benefits of rosemary in burns may be partly attributed to its anti-inflammatory property.

### 4.3 UV-irradiated skin

The metalloproteinases induced by ultraviolet may degrade collagen and thin the skin layer, leading to a loss of skin elasticity and wrinkle formation (Rijken and Bruijnzeel, 2009). Animal models and *in vitro* models suggested associated change in the UV-irradiated areas or cells following treatment with rosemary extracts. 4% and 10% rosemary hexane extract lipid nanocapsule-based mucoadhesive gels were previously tested in a UVB-irradiated rat model, showing improved skin contact, permeation, bioavailability, epidermal and dermal histological features while decreasing the level of inflammatory and wrinkling markers compared to controls (Ibrahim et al., 2022). In a UVB-irradiated mouse model, the anti-wrinkle property and skin permeability of topically applied rosemary extract was enhanced by encapsulation of 20 mg rosemary extract in transferosomes (Ezzat et al., 2016). More specifically, as the major antioxidative components of rosemary extract, carnosol treatment has yielded some beneficial effects in UVB-induced inflammatory skin injuries, including a significant reduction in IgE and proinflammatory cytokines compared to untreated group exposed to UVB, which is in association with the inactivation of STAT3 (Yeo et al., 2019), while CA displayed a protective effect in UVA-irradiated human skin fibroblasts (Offord et al., 2002). When treating the human keratinocytes HaCaT (a spontaneously immortalized cell line) with a combination of rosemary extracts (especially diterpenes and citrus flavanone aglycones) after UVB radiation, some molecular events related to skin photodamage, such as intracellular ROS generation and DNA damage, were significantly attenuated (Vostalova et al., 2010; Perez-Sanchez et al., 2014; Sanchez-Marzo et al., 2020).

The photoprotective potential of rosemary extracts has also been demonstrated in human studies (Perez-Sanchez et al., 2014). However, data from patients is still lacking, and more clinical data is required draw a conclusion whether dietary polyphenols provide a skin photoprotective effect further than the daily consumption of topical sunscreens.

## 5 Conclusion and future directions

In conclusion, the existing body of evidence indicated that rosemary extract and essential oil present a variety of medicinal properties including antimicrobial, anti-inflammatory, antioxidative, analgesic and collagen-promoting properties, which contribute to their potentials in promoting the healing process of a variety of wounds and UV-irradiated skin. However, the contents and quality status of rosemary-related products may be problematic due to the unstandardized preparation and unstable properties. In addition, many of these effects revealed are based on *in vitro* studies and animal studies, and only a limited number of clinical studies have been performed. Considering the differences in skin structure, immune systems and wound healing process between humans and animals, more randomized controlled clinical trials on larger cohorts are needed in the future. Moreover, further research and clinical trials are warranted to fully understand the mechanisms of action of this natural remedy, and to optimize the standardized preparation methods, dose, duration, and form of application for clinical use of rosemary-derived products in wound care, to offer a complementary therapy to enhance the healing process, improve the quality of life and reduce the medical expenditures.
